# Treatment with 3-Aminobenzamide Negates the Radiofrequency-Induced Adaptive Response in Two Cell Models

**DOI:** 10.3390/ijerph16152768

**Published:** 2019-08-02

**Authors:** Anna Sannino, Olga Zeni, Stefania Romeo, Maria Brigida Lioi, Maria Rosaria Scarfì

**Affiliations:** 1 Institute for Electromagnetic Sensing of the Environment (IREA), Italian National Research Council (CNR), 80124 Naples, Italy; 2 Department of Science, University of Basilicata, 85100 Potenza, Italy

**Keywords:** radiofrequency, human lymphocytes, V79 cells, micronuclei, adaptive response, 3-aminobenzamide

## Abstract

In previous investigations, we demonstrated that pre-exposure of different cell cultures to radiofrequency fields can reduce the damage induced by genotoxic agents, an effect resembling the so-called adaptive response. In this study, we pre-exposed human peripheral blood lymphocytes and Chinese hamster lung fibroblast cell line to 1950 MHz, UMTS (Universal Mobile Telecommunication System) signal, for 20 h, and then treated cultures with Mitomycin-C. After confirming the induction of an adaptive response in terms of the reduction of micronuclei formation, we observed that such a response was negated by treatments with 3-aminobenzamide. Since 3-aminobenzamide is an inhibitor of poly (ADP-ribose) polymerase enzyme, which is involved in DNA repair, these results support the possible involvement of DNA repair mechanisms in radiofrequency-induced adaptive response.

## 1. Introduction

The adaptive response (AR) is a universal response of cells to very small doses of ionizing radiation or chemicals, which is manifested by an increased resistance to the damaging effects of higher doses of the same, or other, physical or chemical agents. A large number of in vitro and in vivo studies dealing with the mechanisms behind this phenomenon, suggest that AR is a complex reaction involving a wide range of cellular functions, including cell repair systems [1]. However, a universal mechanism of interaction has not yet been acknowledged [2,3].

Over the last ten years, our research group has repeatedly observed, in in vitro experiments, a protective effect induced by radiofrequency (RF) pre-exposure, referred to as RF-induced AR. Such protection has been detected in several cell types, against several chemical and physical agents, and by analyzing several cellular endpoints. The phenomenon has been characterized with respect to the exposure conditions: 900 MHz and 1950 MHz, different signal modulations, and bandwidth at specific absorption rates (SAR) ranging from 0.15 to 1.25 W/kg, with effective conditions depending on the cell type investigated [4,5,6,7,8,9,10]. Independent research groups have reported similar observations in in vitro and in vivo experiments, under different exposure conditions, in different biological models, and by addressing several biological endpoints. These studies were reviewed and gaps in knowledge were identified [11].

The mechanisms underlying the RF-induced AR need to be investigated, and recent papers suggest the potentiation of oxidative stress defenses and the involvement of DNA repair. In particular, we recently demonstrated that pre-exposure to 1950 MHz, Universal Mobile Telecommunication System (UMTS) signal, reduced menadione-dependent DNA oxidative damage in human neuroblastoma cell cultures by enhancing antioxidant scavenging efficiency and restoring DNA repair capability [9]. This observation is consistent with in vitro and in vivo results reported by other authors [12,13]. Moreover, He and colleagues demonstrated the involvement of poly (ADP-ribose) polymerase-1 (PARP-1) [14,15]. PARP is a family of nuclear enzymes with a key role in several cellular functions, including the repair of DNA strand breaks and genomic instability [16,17,18,19].

In this study, primary human peripheral blood lymphocytes (HPBL), and Chinese hamster lung fibroblasts (V79) were exposed to 1950 MHz electromagnetic field, UMTS signal, for 20 h, at SAR of 0.3 and 1.25 W/kg, respectively, and subsequently treated with mitomycin-C (MMC, a well-known DNA damaging agent), in the presence and in the absence of 3-aminobenzamide (3AB), an inhibitor of PARP. Following treatments, cells were tested for DNA damage by applying the cytokinesis-block micronucleus (MN) assay, a well-known cytogenetic test to evaluate chromosomal damage [20].

## 2. Materials and Methods

### 2.1. RF Exposure and Dosimetry

A well-established RF signal generation and exposure system was used for sample exposure under strictly controlled environmental and electromagnetic conditions, and with high efficiency and uniformity of the SAR distribution in the biological samples. Details on design, realization, and characterization can be found in our previous papers [6,21,22]. Briefly, the signal generation and conditioning chain was composed by a RF signal generator (Agilent, E4432B, Santa Clara, CA, USA), a microwave amplifier (Microwave Amplifier, AM38A-0925-40-43, Bristol, England), a -6 dB power splitter (HP, 11667A, Palo Alto, CA, USA) and two bidirectional power sensors (Rohde and Schwarz, NRT-Z43, Munich, Germany). The exposure chambers consisted of two identical, twin, short-circuited, rectangular waveguides (WR430, 350 mm long, Neyron, France), which were connected to the power sensors by a coaxial adapter (Maury Microwave R213A2, VSWR: 1.05, Montclair, CA, USA) at the feeding end. A third, identical waveguide was used for sham exposure without RF transmission. The three waveguides were housed in the same commercial cell culture incubator (Forma Scientific, Model 311, Freehold, NJ, USA), which maintained a temperature of 37 ± 0.5 °C with a 95% air and 5% CO_2_ atmosphere. In each waveguide, up to four cell cultures, contained in 35 mm Petri-dishes (Corning, catalogue n. 430165, New York, USA), can be arranged by using a four-layer plexiglass stand, and exposed simultaneously to two different SAR values. A SAR ratio of 4:1 was obtained between the central and the distal positions of the stand, and both the efficiency and uniformity of SAR distribution in all samples were above 70% [6,21,22]. In this investigation, SAR values of 0.3 W/kg and 1.25 W/kg were used for HPBL and V79 cells, respectively. Therefore, HPBL cultures were arranged in the distal positions, where the SAR was 0.3 W/kg, whereas dummy cultures, i.e., Petri-dishes filled with culture medium, were arranged in the central positions. In the case of V79 cells, experimental cultures were placed in the central positions, where the SAR was 1.25 W/kg, and dummy cultures were placed in the distal positions of the stand. In both cases, for RF exposure, one of the two powered waveguides was used for samples to be treated with 3AB, and the other for samples that did not undergo 3AB treatment. During exposures, the power level in each waveguide was continuously monitored and adjusted to the required SAR, and the temperature inside cell cultures was measured and recorded using a fiber optic thermometer (FISO Technologies, FisoUMI4, Quebec, Canada), to ensure that variations remained within the accuracy range of the instrument (37 ± 0.3 °C).

### 2.2. Experimental Protocol and Culture Setup

The experiments on human lymphocytes were performed in accordance with high standards of ethics and approved by the Ethics Committee of the National Institute of Cancer, G. Pascale Foundation (Naples, Italy) under the ethical approval code n. 541. Donors were informed about the aim of the study and they provided written consent. Isolated lymphocytes were obtained from buffy coats from three, non-smoking healthy male donors (age range 24–34 years) through Lymphoprep density gradient centrifugation (LymphoprepTM, Axis-Shield, Oslo, Norway) [23]. For the experiments, 7 × 10^5^ cells/mL were cultured for 72 h in 3 mL RPMI (Roswell Park Memorial Institute) 1640 medium, supplemented with 15% fetal bovine serum (FBS), 1% L-glutamine, 1% penicillin/streptomycin, and 1% phytohemaglutinin (PHA) as mitogen to stimulate T lymphocytes to enter the cell cycle.

V79 cell line was purchased from Sigma (St. Louis, MO, USA), cultured in DMEM (Dulbecco’s Modified Eagle’s Medium) supplemented with 10% FBS, 1% L-glutamine, and 1% penicillin/streptomycin, and maintained in an exponential growth phase by sub-culturing two times a week by typsinization. For the experiments, 5 × 10^4^ cells/mL were seeded in 3 mL of complete medium in Petri dishes 8 h before treatments, and grown for a total of 48 h. Three independent experiments were carried out. Cell culture reagents, for both cell types, were purchased from Biowhittaker (Verviers, Belgium).

**Figure 1:**
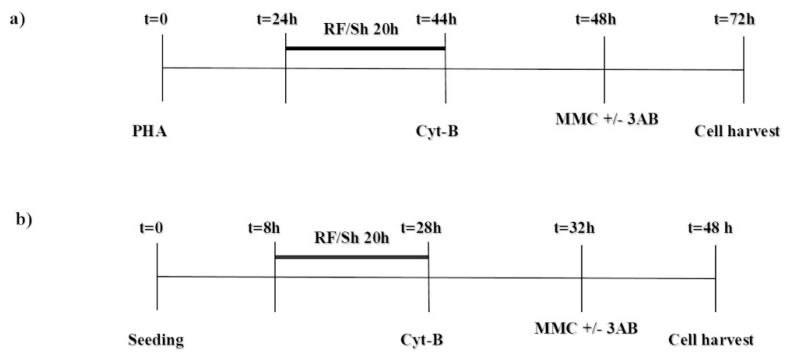
Experimental procedures adopted to treat cell cultures with radiofrequency/sham (RF/Sh) and mitomycin-C (MMC). Timing for 3-aminobenzamide (3AB) treatment (2 mM) is also included. Human peripheral blood lymphocyte (HPBL) treatment: 0.3 W/kg specific absorption rate (SAR); Cytochalasin-B (Cyt-B) 6 µg/mL; MMC 100 ng/mL (panel **a**). V79 cell treatment: 1.25 W/kg SAR; Cyt-B 3 µg/mL; MMC 500 ng/mL (panel **b**).

For both cell types, each experimental run included two groups of 6 cultures, one in the presence and one in the absence of 3AB, as follows: (i) untreated control; (ii) MMC, (iii) RF, (iv) RF + MMC, (v) sham (Sh), (vi) Sh + MMC. MMC (Sigma, St. Louis, MO, USA) was dissolved in sterile distilled water, and given at 48 h (100 ng/mL) or 32 h (500 ng/mL) after seeding, for HPBL and V79 cells, respectively. 3AB (Sigma, St. Louis, MO, USA) was dissolved in dimethyl-sulfoxide (DMSO, Lab-Scan Analytical Science, Dublin, Ireland), and given at 48 h (HPBL) or 32 h (V79 cells) at a final concentration of 2 mM, according to the procedure reported in the literature for several cell types [15,24]. To block cytokinesis, cytochalasin-B (Sigma, St. Louis, MO, USA; dissolved in DMSO) was added at a final concentration of 6 µg/mL at 44 h after PHA stimulation in HPBL cultures, and at 3 µg/mL at 28 h after seeding in V79 cultures. A schematic representation of the experimental procedures is presented in Figure 1.

At the end of the total culture period, HPBL were collected and microscope slides were made up as previously reported [6]. The same protocol, with minor modification, was applied for V79 cells [8]. Cells on slides were fixed (80% methanol solution) and stained (10% Giemsa solution), and coded slides were examined at 1000× magnification. For each treatment, 2000 binucleate (BN) cells were examined to record the incidence of MN. On the same slides, 1000 cells were classified according to the number of nuclei, and the proliferation index (PI), an index of cytotoxicity, was derived as [M1 + 2M2 + 3 (M3 + M4)]/N, where M1 to M4 represent the number of cells with 1 to 4 nuclei, and N is the total number of cells scored [25]. All data were decoded after completing the microscopic examination of slides.

To account for the inter-donor variability in the case of HPBL, and the day-to-day variation in the case of V79 cells, the results of the MN assay were analyzed by means of the chi-square (χ^2^) test. PI data were evaluated by applying the unpaired Student’s *t* test. Excel (Microsoft Office, Redmond, Washington, USA) software was used for the analysis, and statistical significance was set at *p* < 0.05.

## 3. Results and Discussion

The cell models chosen in this study have been widely employed in research on DNA damage and repair by genotoxic agents, including investigations on AR [8,26,27]. Moreover, HPBL are primary human blood cells, accounting for human exposure to electromagnetic fields in a real-life scenario, and V79 is a stabilized, fibroblast cell line from rodents. Well-established experimental protocols are available for both cell models. To account for the different cell cycle durations and sensitivity to chemicals of the two cell types, different experimental procedures were adopted (Figure 1). In addition, in our earlier papers, 20 h RF exposure and SAR levels of 1.25 W/kg and 0.3 W/kg were effective in inducing AR in HPBL [6] and V79 cells [8], and therefore were used in the present study.

**Figure 2:**
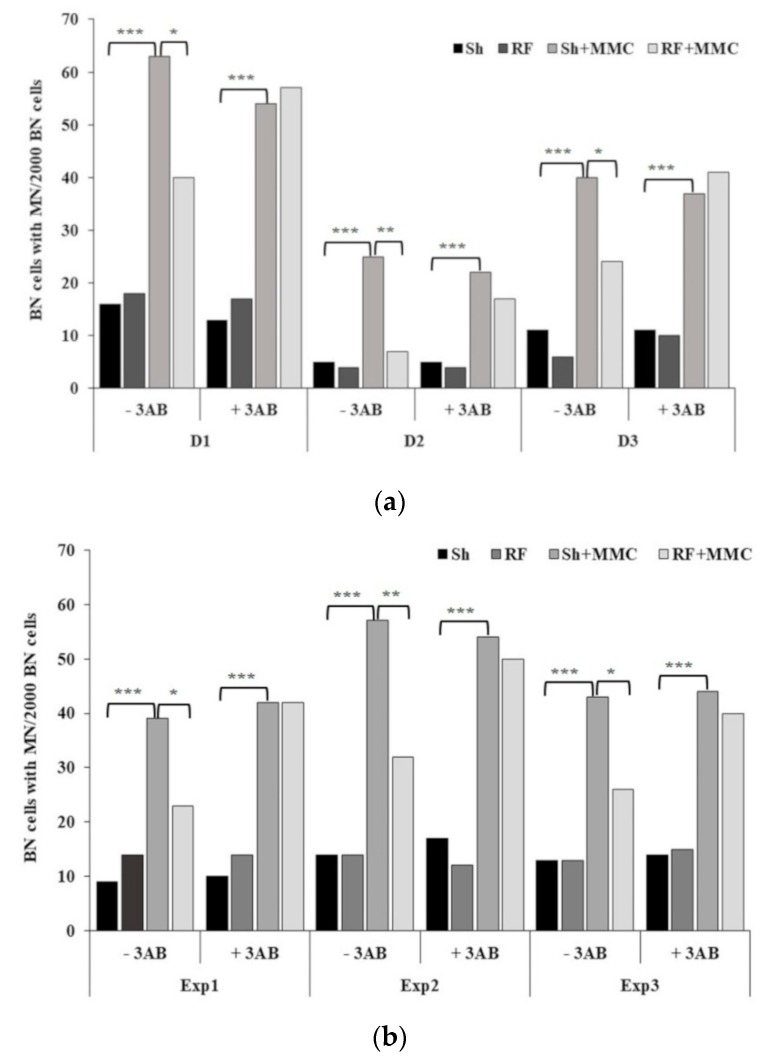
Micronuclei (MN) frequency in 2000 binucleate (BN) cells on (**a**) human peripheral blood lymphocytes from three donors (D1–D3), and (**b**) in three independent experiments on V79 cells (Exp1–3). For each cell type, results of Sh, RF, Sh + MMC, and RF + MMC treatments are reported, in the absence and in the presence of 3AB. χ^2^ test: * *p* < 0.05; ** *p* < 0.01; *** *p* << 0.01.

The results obtained in the HPBL and V79 cultures are reported in Figure 2, in panels (a) and (b), respectively. In all the experimental runs, there was no statistically significant difference in MN formation between control and sham-exposed cultures, in the presence and absence of MMC (*p* > 0.05, data not shown). Therefore, sham-exposed cultures (Sh and Sh + MMC) were considered as the most appropriate reference controls in all experiments. RF exposure never induced MN formation in either cell type (Sh vs. RF: *p* > 0.05), whereas, as expected, MMC treatment induced a statistically significant MN increase (Sh + MMC vs. Sh: *p* << 0.01) in all donors/experiments. These observations also held true in the presence of 3AB, indicating that the addition of 3AB had no effect on MN induction.

Concerning the adaptation protocol, in the absence of 3AB, in both cell types, exposure to RF + MMC resulted in a significant decrease in MN incidence compared to cells treated with MMC alone (RF + MMC vs. Sh + MMC: *p* < 0.05), with the decrease ranging between 37% and 72% in the case of HPBL, and between 39% and 44% in the case of V79, confirming our previous findings [6,8]. Interestingly, in both cell types, RF-induced AR was negated in the presence of 3AB (*p* > 0.05, RF + MMC + 3AB vs. Sh + MMC + 3AB), suggesting the involvement of DNA repair enzymes in eliciting AR, and indicating that the phenomenon may not be dependent on the cell model. It should be noted that in HPBL from the three donors involved in this study, a high variability in MN frequency, both spontaneous and MMC-induced, was recorded. This finding is consistent with the variability reported by other authors in ionizing radiation-induced AR, and this may in part be genetically controlled [28,29].

**Figure 3:**
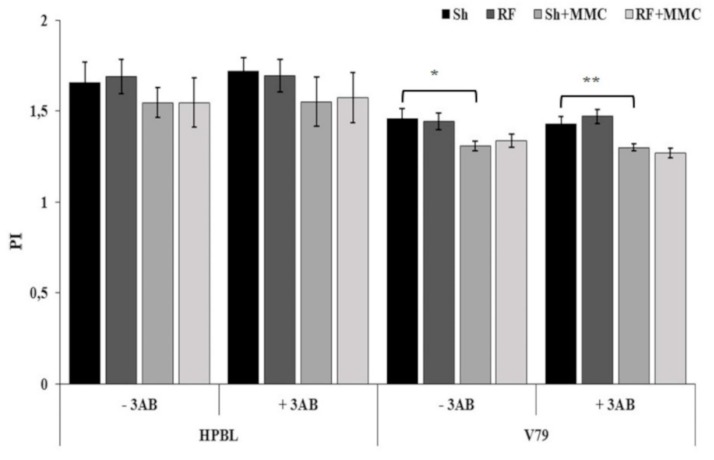
Cytotoxicity, expressed as the proliferation index (PI), in 1000 HPBL and V79 cells. For each cell type, results of Sh, RF, Sh + MMC, and RF + MMC treatments are reported, in the absence and in the presence of 3AB. Each data point represents the mean ± SD of three independent experiments. Unpaired Student’s *t* test: * *p* < 0.05; ** *p* < 0.01.

PI results were unaffected in HPBL for all the treatments, irrespective of 3AB addition, whereas a slight but statistically significant decrease was recorded in V79 cells when Sh + MMC samples were compared to Sh samples, both in the absence and in the presence of 3AB (*p* < 0.05). These results are consistent with our previous findings [6,8] and are presented in Figure 3.

In a recent paper, direct evidence has been provided for the involvement of PARP-1 in RF-induced AR. He and colleagues exposed mouse bone marrow stromal cells to 900 MHz, continuous wave, at 120 µW/cm^2^ for 3h/day for five days, and a significant increase in PARP-1 mRNA expression and its protein level was detected with respect to sham controls [14]. In a subsequent study, the authors found that, in the same experimental conditions, the increase in PARP-1 was reduced after 3AB treatment. An increase in PARP-1 was also detected in cells exposed to RF and Ɣ rays, compared to samples exposed to Ɣ rays alone, while it was negated by 3AB treatment [15]. The overall results indicate that PARP-1 has a role in RF-induced AR, as indicated by direct (PARP-1 mRNA expression and its protein level) and indirect (3AB treatment) measurements.

The possible involvement of PARP, here demonstrated by indirect measurements through 3AB treatment, confirms the results reported by He and colleagues and extends them to different cell types, subjected to different experimental protocols in terms of electromagnetic characteristics, exposure duration, DNA damaging agent, and the endpoint investigated. Moreover, the results presented here add to our recent findings where the involvement of 8-oxoguanine DNA glycosylase (OGG1), a critical DNA repairing enzyme, was demonstrated in the induction of AR in human neuroblastoma cell cultures pre-exposed to RF (1950 MHz, UMTS signal), and then treated with menadione, an oxidative DNA damage inducer. Specifically, RF pre-exposure was able to abolish the menadione-induced down-regulation of OGG1, thus restoring DNA repair capability [9].

## 4. Conclusions

The results reported in the present investigation indicate that RF-induced AR, in two cell types, is negated by the addition of 3AB, an inhibitor of PARP. Our findings further highlight DNA repair mechanisms as one of the plausible candidates in eliciting RF-induced AR, although further investigation is required to give insight into the action mechanism.
